# A Tri-Stable Piezoelectric Vibration Energy Harvester for Composite Shape Beam: Nonlinear Modeling and Analysis

**DOI:** 10.3390/s20051370

**Published:** 2020-03-02

**Authors:** Xuhui Zhang, Meng Zuo, Wenjuan Yang, Xiang Wan

**Affiliations:** 1 College of Mechanical Engineering, Xi’an University of Science and Technology, Xi’an 710054, China; yanggwenjuan@163.com (W.Y.); wx@xust.edu.cn (X.W.); 2 Shaanxi Key Laboratory of Mine Electromechanical Equipment Intelligent Monitoring, Xi’an 710054, China

**Keywords:** piezoelectric vibration energy harvesting, composite shape beam, nonlinear magnetic force, nonlinear restoring force, harmonic balance

## Abstract

To reveal the nonlinear mechanism of the tri-stable piezoelectric vibration energy harvester based on composite shape beam (TPEH-C) and its influence on the system response, the nonlinear restoring force and the nonlinear magnetic force are discussed and analyzed in this paper. The nonlinear magnetic model is acquired by using equivalent magnetizing current theory, and the nonlinear resilience model is obtained by fitting experimental data. The corresponding distributed parameter model based on generalized Hamiltonian variation principle has been established. Frequency response functions for the TPEH-C are derived according to harmonic balance expansion, and the influence of different magnet distances and different excitation accelerations on the response amplitude and bandwidth of the TPEH-C are investigated. More importantly, the correctness of the theoretical analysis is verified by experiments. The results reveal that the spectrum of composite beam shows hard characteristic and the depth of potential well is changed, which provides a new way to ameliorate the potential well of the TPEH-C. A suitable magnet distance enables the TPEH-C to improve the response amplitude and the effective frequency range. The results in this paper have a theoretical guiding significance for the optimal design and engineering application of the TPEH-C.

## 1. Introduction

Microelectronics and wireless sensing technology support the rapid growth of on-line monitoring technology for coal mine machine. However, the energy supply of the sensor nodes is the bottleneck in its development [1,2]. Environmental energy harvesting technology is widely used in power supply and condition monitoring of micro-electronic equipment, among which vibration energy harvesting has become a research hotspot because it is not affected by position and environment [3,4,5,6,7]. Thus, it is expected to alleviate the trouble of wiring and power supply for on-line monitoring of coal mine equipment by using the piezoelectric energy harvester to power a wireless monitoring node [8].

Generally, environmental excitation is broad band and multi-directional. In order to adapt the energy harvester traps to these characteristics, a lot of work in structural design and theoretical research has been done [9,10,11,12]. A kind of piezoelectric energy harvester with clamped–clamped beams structure is introduced in [13]. This structure has a high frequency response, and broad band during the frequency sweep test. Chen et al. [14] investigate a structure with one-dimensional phononic piezoelectric cantilever beams, which improve the response bandwidth by adjusting the mass. Fu et al. [15] designed a piezoelectric vibration energy harvester for low-speed and broad band, and the influence of different structural parameters on the output is analyzed. Paknejad et al. [16] derived the steady-state response of piezoelectric energy harvester for various thin multilayer composite beams under harmonic excitation, and the relationship between the natural frequency and damping ratio has been studied. To conform to the multi-directional environment, a flexible longitudinal zigzag structure is designed in [17,18]. This structure is capable of effective energy conversion in a wide range of excitation directions. Yang et al. [19] proposed that arched structures have more advantages over straight beams in the energy collection. Zhang et al. [20,21] investigated a new piezoelectric transducer for composite beam structure based on magnetic coupling. The arc-shaped beam can satisfy the multi-direction requirement of ambient vibration source, so it has advantages in the adaptability of environmental vibration.

At present, the frequency-extension method using nonlinear behavior has grown up to be a hot topic of energy harvester research. Wei et al. [22] introduced a scissor-like structure energy harvester, which nonlinear damping improves the vibration energy collection performance. The linear and nonlinear models of the U-shaped vibration-based piezoelectric energy harvester (U-VPEH) are compared in [23]. It proves that the nonlinear U-VPEH shows superior performance in energy collection. Erturk and Inman [24,25] verified experimentally that the nonlinear transition behavior induces high-energy interwell oscillations of the bi-stable energy harvester under harmonic excitation, which greatly improves the energy harvesting performance. The dynamic response transformation between hardening nonlinearity and softening nonlinearity makes the energy harvester more adaptable to environment [26]. The wideband tri-stable energy harvester based on nonlinear magnetic field has attracted much attention since it was offered [27,28]. The relationship between potential well depth and tri-stable energy harvester performance was numerically and experimentally studied in [29]. Cao et al. [30] investigates the load resistance of tri-stable energy harvesting from real human motion excitation indicate that there always exists an optimum load resistance to maximize the average output power. Zhou et al. [31] derived the nonlinear response characteristics of the asymmetric tri-stable energy harvester to improve energy harvesting performance for more general applications. Wang et al. [32] proved that the tri-stable energy harvester possesses a wider potential well width and a lower barrier height, which greatly extends the operating frequency band. The tri-stable configuration has been demonstrated to enable piezoelectric energy harvester to generate electricity in a wider frequency range, and improve energy harvesting efficiency [33,34,35].

On-line monitoring of coal mining equipment needs a kind of energy harvesting device to realize self-power supply, which can adapt to the multi-direction and broadband characteristics of equipment. Compared with other energy harvesting structures, the tri-stable energy harvester is relatively simple, and has been proved to have a wider spectrum characteristic than the monostable harvester in some excitation conditions. It can improve the energy harvesting performance, which has some advantages in the power supply of equipment monitoring nodes. However, in general tri-stable energy harvesters, the cantilever beam is a linear beam structure. The research on the linear-arch composite beam structure is less, especially the nonlinear restoring force and its influence on the tri-stable energy harvester have not been reported.

Aiming at enhancing energy harvesting from multi-direction and broadband environmental excitations, the tri-stable piezoelectric vibration energy harvester based on composite shape beam (TPEH-C) is proposed in this paper. The nonlinear magnetic force model of the system is established using the equivalent magnetizing current theory, and the nonlinear restoring force is fitted by the experimental data. The distributed parameter model based on generalized Hamiltonian variation principle is established. Frequency response functions of the TPEH-C are derived according to the harmonic balance method, and the influence of different magnet distances and different excitation accelerations on the response amplitude and bandwidth of the TPEH-C are investigated. Finally, the theoretical analysis is verified by experiments.

## 2. Theoretical Modeling

The distributed parameter method based on Hamiltonian principle is more general than the lumped parameter method because it considers the distribution quality, which was used in many studies. However, the restoring force of the linear-arch composite beam structure is not linear and needs to be studied and discussed to ensure the accuracy of the model. In this section, the restoring force and the magnetic field of the beam are first separately modeled and discussed to ensure more accurate models being obtained. Then, according to the Hamiltonian principle, the dynamic model of the system is obtained.

### 2.1. The TPEH-C

**Figure 1:**
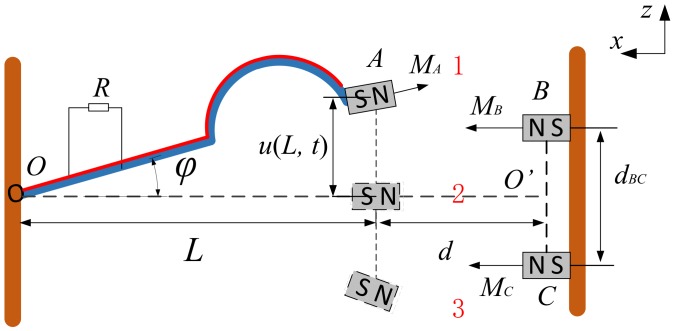
The schematic diagram of the TPEH-C.

In order to solve the problem of node power supply for on-line monitoring of coal mine machineries, piezoelectric vibration energy harvesting technique is proposed. The TPEH-C can adapt to the multi-direction and broadband external excitations. Due to the limited size of the joint power supply equipment, the composite beam structure can provide more piezoelectric material area than the linear structure. The piezoelectric vibration energy harvester for the linear-arch composite beam is illustrated in Figure 1. The TPEH-C comprises of a base, a linear-arch composite beam, a Polyvinylidene Fluoride (PVDF) piezoelectric layer, a tip magnet attachment and two external magnets. The length of the linear-arch composite beam in the *x*-axis direction is *L*, and the piezoelectric layer is completely attached to the matrix of the beam. The distance between the tip magnet and the middle of two external magnets is *d*, and between external magnets is $d_{BC}$. When the external excitation along the *z* axis acts on the beam, it vibrates and deforms. PVDF, adhered to the surface of the composite beam, converts the vibration energy into electrical energy due to the direct piezoelectricity. It is assumed that the relative vibration displacement of the beam is $u\left(X,t\right)$. There are two nonlinear factors in the TPEH-C: one is the nonlinear restoring force due to the linear-arch composite beam structure; the other is the nonlinear magnetic force generated by the external magnet in the moving space of the beam.

### 2.2. Modeling of Nonlinear Restoring Force

Unlike the linear restoring force of a typical straight beam, the restoring force is nonlinear in the composite beam because of the arch shape structure. Therefore, the nonlinear restoring force should be analyzed. A dynamometer (YLK-10) was used to measure the restoring force of the composite beam in the *z*-axis direction. The composite beam is mounted on the fixture, and the end of the beam is pushed by dynamometer to record the measured data of displacement and force. After measured several times and averaging, the curve fitting method is used to obtain the nonlinear recovery force expressions

(1) $$F_{r}=s_{2}u{\left(L,t\right)}^{3}+s_{1}u\left(L,t\right)$$

where,$\;s_{2}$ and $s_{1}$ are polynomial coefficients obtained by experiment. $u\left(L,t\right)$ is the displacement of the beam along the *z*-axis at *t.*

**Figure 2:**
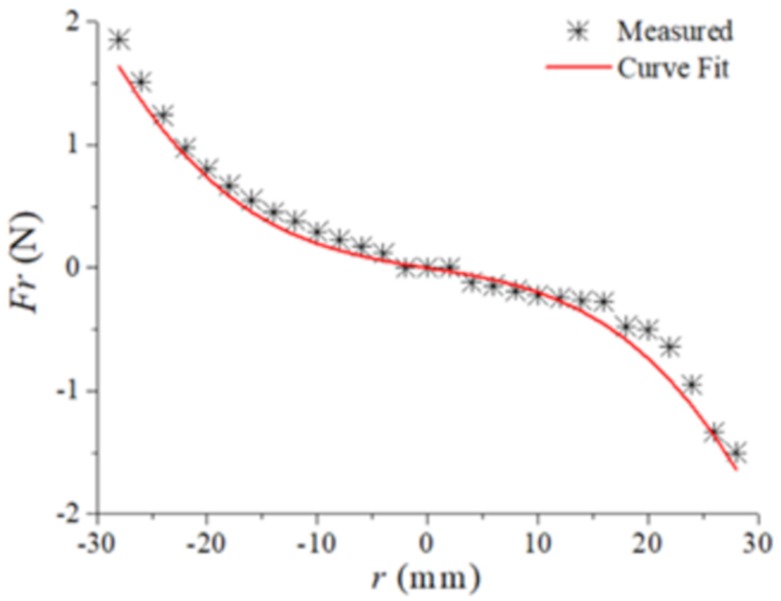
The nonlinear restoring force of linear-arch composite beam.

Figure 2 is the experimental measurement and curve fitting results of nonlinear restoring force of linear-arch composite beam. The trinomial coefficients $s_{2}=-41,963.4\;N/m^{3}$ and one-time coefficients $s_{1}=-18.29\;N/m$. It can be observed in the diagram that the restoring force of the cantilever beam presents a curve due to the existence of the arched part. Setting u=0 as the equilibrium position, and the measurement results are slightly asymmetrical. This is explained by the fact that when the curvature of the arch part becomes larger in a certain deformation range, the force required smaller than the curvature becomes smaller.

### 2.3. Modeling of the Nonlinear Magnetic Force

**Figure 3:**
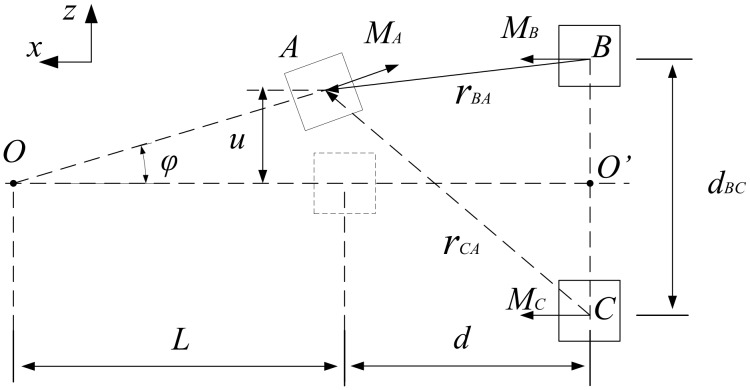
The nonlinear magnetic force of the TPEH-C.

Building an accurate theoretical model is the basis of theoretical research. Compared with the magnetic dipole model, the magnetizing current method describes more accurately when the magnetic distance is not large [36]. The position and geometry of the magnet in the TPEH-C are given in Figure 3. The midpoint *O’* between magnet B and magnet C is taken as coordinate origin. The length, width and height of the three magnets are $l_{a}$, $l_{b}$, $l_{c}$, $h_{a}$, $h_{b}$, $h_{c}$, $w_{a}$, $w_{b}$, $w_{c}$, respectively. When the magnet A is in the horizontal position, the distance from the point *O’* is *d*. The length from A to O is $L+l_{a}/2$, and the angle between the cantilever beam and horizontal direction is φ. From the geometric relation,$\;sin\varphi =\frac{u}{L+l_{a}/2}$ and $\cos\varphi =\sqrt{1-sin^{2}\varphi }$ can be obtained. The $u\left(L,t\right)$ is simply written as u.

Assuming that the magnetic field generated by the magnet is evenly distributed in space. According to the magnetizing current method [37], the magnetic forces on magnet A in *z* direction due to the magnetic field produced by magnets B and C. The magnetic force is given by:

(2) $$F_{m}=\mu_{0}M_{A}SHb_{2}\left(d-\frac{h_{a}}{2}\cdot sin\varphi ,0,u-\frac{h_{a}}{2}\cdot cos\varphi -\frac{d_{BC}}{2}\right)-\ldots \;Hb_{1}\left(d+\frac{h_{a}}{2}\cdot sin\varphi ,0,u+\frac{h_{a}}{2}\cdot cos\varphi -\frac{d_{BC}}{2}\right)+\ldots \;Hc_{2}\left(d-\frac{h_{a}}{2}\cdot sin\varphi ,0,u-\frac{h_{a}}{2}\cdot cos\varphi +\frac{d_{BC}}{2}\right)-\ldots \;Hc_{1}\left(d+\frac{h_{a}}{2}\cdot sin\varphi ,0,u+\frac{h_{a}}{2}\cdot cos\varphi +\frac{d_{BC}}{2}\right)$$

where, *S* is the top or bottom surface area of magnet A. $\;Hb_{1}$ and $Hb_{2}$ are the magnitudes of the magnetic field strength generated by magnet B at the centers of magnet A’s top and bottom surfaces in x direction. Similarly, $Hc_{1}$ and $Hc_{2}$ are the magnitudes of the magnetic field strength generated by magnet C.

The expressions are:

(3) $$Hb=\frac{M_{B}}{4\pi }[tan^{-1}\left(\frac{z_{b1}y_{b1}}{X\sqrt{z_{b1}{}^{2}+y_{b1}{}^{2}+X^{2}}}\right)+tan^{-1}(\frac{z_{b2}y_{b2}}{X\sqrt{z_{b2}{}^{2}+y_{b2}{}^{2}+X^{2}}})-\;tan^{-1}\left(\frac{z_{b2}y_{b1}}{X\sqrt{z_{b2}{}^{2}+y_{b1}{}^{2}+X^{2}}}\right)-tan^{-1}\left(\frac{z_{b1}y_{b2}}{X\sqrt{z_{b1}{}^{2}+y_{b2}{}^{2}+X^{2}}})\right]$$

where, $z_{b1}=u+\frac{h_{B}}{2},z_{b2}=u-\frac{h_{B}}{2},y_{b1}=y+\frac{w_{B}}{2},y_{b2}=y-\frac{w_{B}}{2}$.

(4) $$Hc=\frac{M_{C}}{4\pi }[tan^{-1}\left(\frac{z_{c1}y_{c1}}{X\sqrt{z_{c1}{}^{2}+y_{c1}{}^{2}+X^{2}}}\right)+tan^{-1}(\frac{z_{c2}y_{c2}}{X\sqrt{z_{c2}{}^{2}+y_{c2}{}^{2}+X^{2}}})-\ldots \;tan^{-1}\left(\frac{z_{c2}y_{c1}}{X\sqrt{z_{c2}{}^{2}+y_{c1}{}^{2}+X^{2}}}\right)-tan^{-1}\left(\frac{z_{c1}y_{c2}}{X\sqrt{z_{c1}{}^{2}+y_{c2}{}^{2}+X^{2}}})\right]$$

where, $z_{c1}=u+\frac{h_{c}}{2},z_{c2}=u-\frac{h_{c}}{2},y_{c1}=y+\frac{w_{c}}{2},y_{c2}=y-\frac{w_{c}}{2}$.

Using the curve fitting method, the magnetic expression is reduced to a polynomial expression about displacement u(L,t):

(5) $$F_{m}=k_{3}u^{5}\left(L,t\right)+k_{2}u^{3}\left(L,t\right)+k_{1}u\left(L,t\right)$$

where, the coefficients $k_{1}$, $k_{2}$ and $k_{3}$ are obtained by curve fitting the results of the magnetic force $F_{m}$ calculation.

**Figure 4:**
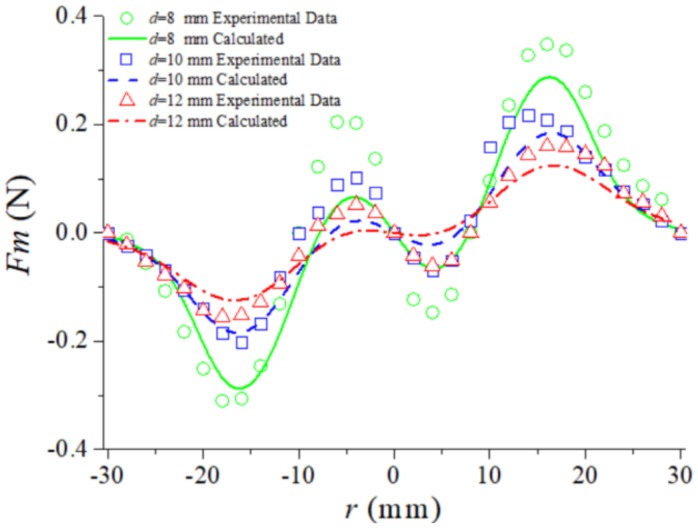
The comparison of the magnetic force between calculations and experimental measurement.

**Figure 5:**
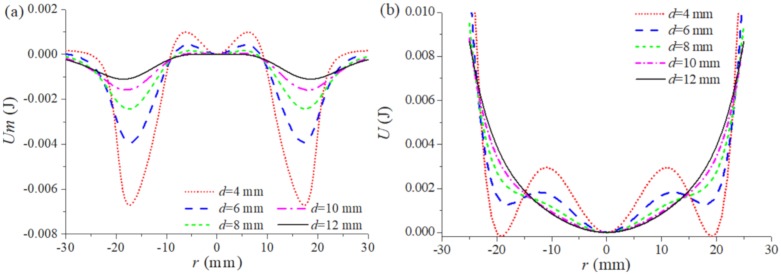
(**a**) The magnetic potential functions at different magnet distances; (**b**) The nonlinear restoring force potential functions of TPEH-C.

To verify the accuracy of the nonlinear magnetic force model, a dynamometer was used to measure the magnetic force of three groups of different magnetic distances *d*. The two magnets were fixed on the base and they have a center distance of $d_{BC}=20\;mm$. The free magnet was fixed on the dynamometer’s push rod. At a different distance *d*, we can use the dynamometer to push the free magnet along the *z*-axis direction, and record the displacement and the force. The experimental measurements are compared with the magnetic curves obtained from the model, as shown in Figure 4. It can be found from the diagram that the theoretical calculation results are in accordance with experimental results, which means that the nonlinear magnetic field model is established in this paper accurately. The reason why the magnetic force measurement results are slightly larger than the calculated results is that the force in the *x*-axis direction is neglected in the calculation. As can be seen from the curve in observation Figure 4, the tri-stable magnetic force presents two peaks and two troughs. There are five points of the magnetic force $F_{m}=0$, which constitute the inflection point in the potential energy (as shown in Figure 5).

Figure 5a shows the magnetic potential functions at different magnet distances, and Figure 5b is the nonlinear restoring force potential functions of the TPEH-C. It can be found by comparison that the potential functions in Figure 5a are deeper than in Figure 5b, while the one in the middle is slightly shallower than that in Figure 5b. It proves that the restoring force of the composite beam has an effect on the depth of potential wells of the TPEH-C. The reason for this phenomenon is the interaction between the restoring force and the magnetic force. When the composite beam is in a huge deformed position, where the restoring force and the magnetic force cancel each other out, the potential well becomes shallow. When it in a small deformation position the restoring force and the magnetic force superimpose each other, the potential wells deepen.

In Figure 5b, the moving space of the tip magnet is divided into three parts due to the interaction between the magnetic force and the restoring force. The three steady-state correspond to the three positions depicted in Figure 1. When the magnet distance is *d* = 4 mm, the potential well is the deepest. If the TPEH-C is to produce a considerable motion between wells, it needs to consume a lot of energy over the barrier. Therefore, if the external excitation cannot provide enough energy to ensure that the TPEH-C crosses the barrier, the system can only carry out a small movement in the vibration process. As the magnet distance d increases, the potential well becomes shallower. When the magnet distance *d* > 10 mm, the system is close to the monostable system, at which time there is only one potential well. When the magnet distance *d* = 8 mm or 10 mm, the TPEH-C is reaching a critical point of tri-stable versus monostable state. the TPEH-C not only retains the tri-stable characteristics, but the barrier is also obviously reduced, which is the ideal motion state of the energy harvester system.

### 2.4. Modeling of the TPEH-C

According to the generalized Hamiltonian variational principle, the variation of Lagrange function of the TPEH-C is equal to 0 in any time period $t_{1}$ and $t_{2}$. That is, the system satisfies the equation

(6) $$V.I.=\int_{t1}^{t2}\left[\delta \left(T^{*}-W^{*}\right)+\delta W_{nc}+\delta U_{m}\right]dt=0$$

where δ is the variational symbol. $T^{*}$, $W^{*}$, $W_{nc}$ and $U_{m}$ respectively represent the kinetic, potential energy, virtual work of external forces and magnetic potential.

Suppose $u\left(X,t\right)$ is the displacement of a point on a cantilever along the *z*-axis at *t*, and the beam satisfies the Rayleigh–Ritz principle. The vibrational displacement $u\left(X,t\right)$ of the beam can be discretized into a combination of modes of each order

(7) $$u\left(X,t\right)=\overset{n}{\underset{i=1}{\sum }}\psi_{i}\left(X\right)r_{i}\left(t\right)$$

where $\psi_{i}\left(X\right)$ represents the *i*st-order modal mode functions of the beam. $r_{i}\left(t\right)$ is the modal coordinates. For the boundary conditions where one end is free and one end is clamped, the allowable function can be expressed as:

(8) $$\psi_{i}\left(X\right)=1-cos\left[\frac{\left(2i-1\right)\pi x}{2L}\right]$$

Under low frequency excitations, the vibration of the composite beam is mainly concentrated in the first-order mode. So, only the first-order mode is considered, that is, i=1.

According to Euler–Bernoulli beam theory, piezoelectric constitutive equation and Kirchhoff’s law, the dynamic expression of the TPEH-C is obtained by combining Equations (1), and Equations (5)–(7).

(9) $$M\ddot{r}+C\dot{r}+F_{r}-\theta v-F_{m}=H_{s}\ddot{y}\left(t\right)$$

(10) $$\theta \dot{r}+C_{p}\dot{v}+\frac{v}{R}=0$$

where $F_{r}$ is the nonlinear restoring force of linear-arch composite beam. $F_{m}$ is nonlinear magnetic force. M is the modal mass, and the expression of M is obtained by treating the end block as a concentrated mass.

(11) $$M=\left(\rho_{b}A_{b}+\rho_{p}A_{p}\right)\int_{0}^{L}\psi^{2}\left(X\right)dX+m_{0}\psi^{2}\left(L\right)$$

where C, θ, and $H_{s}$, are respectively the damping, the electromechanical coupling term and the fundamental excitation coefficient.$\;C_{p}$ is the piezoelectric element capacitance, and the external resistance of the system is R

(12) $$C=cA_{b}\int_{0}^{L}\psi^{2}\left(X\right)dX$$

(13) $$\theta =\frac{ze_{31}A_{p}}{h_{p}}\int_{0}^{L}\psi^{\prime \prime }\left(X\right)dX$$

(14) $$H_{s}=\left(\rho_{b}A_{b}+\rho_{p}A_{p}\right)\int_{0}^{L}\psi \left(X\right)dX+m_{0}\psi \left(L\right)$$

(15) $$C_{p}=\frac{\varepsilon_{33}^{S}b_{p}L_{p}}{h_{p}}$$

where, $\rho_{b}$ and $\rho_{p}$ are density of the substrate layer and the piezoelectric layer. $A_{b}$ and $A_{p}$ are cross sectional area of composite beam and piezoelectric layer, respectively. $m_{0}$ is the end block mass.

## 3. Harmonic Balance Solutions

There are many approximate analytical methods for nonlinear systems. However, the harmonic balance method is very effective to analyze the frequency response of nonlinear systems. In this paper, the harmonic balance method is used to obtain the analytical expression of the response of the system under base harmonic excitations. The nonlinear stiffness model and the nonlinear magnetic force model are substituted into Equations (9) and (10)

(16) $$M\ddot{r}+C\dot{r}-k_{3}r^{5}-(k_{2}-s_{2})r^{3}-(k_{1}-s_{1})r-\theta v=H_{s}\ddot{y}\left(t\right)$$

(17) $$\theta \dot{r}+C_{p}\dot{v}+\frac{v}{R}=0$$

In order to analyze the response characteristics of TPEH-C under harmonic excitation, it is assumed that the external excitation is $\ddot{y}\left(t\right)=Acos\left(\omega t\right)$. The steady-state response displacement and voltage of the system are as follows:

(18) $$r\left(t\right)=a_{0}\left(t\right)+a\left(t\right)sin\left(\omega t\right)+b\left(t\right)cos\left(\omega t\right)$$

(19) $$v\left(t\right)=p\left(t\right)sin\left(\omega t\right)+q\left(t\right)cos\left(\omega t\right)$$

where, $a_{0}\left(t\right)$ is a constant term indicating the equilibrium position.

After substituting Equation (18) into Equation (16) and Equation (19) into Equation (17), balancing the terms multiplied by $sin\left(\omega t\right)$, $cos\left(\omega t\right)$ and constant terms, the following equations are obtained:

(20) $$M\ddot{a_{0}}+C\dot{a_{0}}-k_{3}(a_{0}^{5}+5a_{0}^{3}\left(a^{2}+b^{2}\right)+\frac{15}{8}a_{0}{\left(a^{2}+b^{2}\right)}^{2})-(k_{2}-s_{2})(a_{0}^{3}+\ldots \;\frac{3}{2}a_{0}\left(a^{2}+b^{2}\right))-(k_{1}-s_{1})a_{0}=0$$

(21) $$M\left(\ddot{a}-2\dot{b}\omega -a\omega^{2}\right)+C\left(\dot{a}-b\omega \right)-k_{3}a(5a_{0}^{4}+\frac{15}{2}a_{0}^{2}\left(a^{2}+b^{2}\right)+\frac{5}{8}{\left(a^{2}+b^{2}\right)}^{2})-\ldots \;(k_{2}-s_{2})a(3a_{0}^{2}+\frac{3}{4}\left(a^{2}+b^{2}\right))-(k_{1}-s_{1})a-\theta p=0$$

(22) $$M\left(\ddot{b}+2\dot{a}\omega -b\omega^{2}\right)+C\left(\dot{b}+a\omega \right)-k_{3}b(5a_{0}^{4}+\frac{15}{2}a_{0}^{2}\left(a^{2}+b^{2}\right)+\frac{5}{8}{\left(a^{2}+b^{2}\right)}^{2})-\ldots \;(k_{2}-s_{2})b(3a_{0}^{2}+\frac{3}{4}\left(a^{2}+b^{2}\right))-(k_{1}-s_{1})b-\theta q-H_{s}A=0$$

(23) $$\theta \left(\dot{a}-b\omega \right)+C_{p}\left(\dot{p}-\omega q\right)+\frac{1}{R}p=0$$

(24) $$\theta \left(\dot{b}-a\omega \right)+C_{p}\left(\dot{q}+\omega p\right)+\frac{1}{R}q=0$$

The second derivative of the equations $\ddot{a_{0}}=\ddot{a}=\ddot{b}=0$, and first derivative $\dot{a_{0}}=\dot{a}=\dot{b}=\dot{p}=\dot{q}=0$. The expressions of harmonic coefficients  p and  q for a and b can be obtained from Equations (23) and (24), and the frequency response of steady-state displacement can be obtained by substituting them into Equations (20)–(22).

(25) $$k_{3}(a_{0}^{5}+5a_{0}^{3}\eta^{2}+\frac{15}{8}a_{0}\eta^{4})+(k_{2}-s_{2})(a_{0}^{3}+\frac{3}{2}a_{0}\eta^{2})+(k_{1}-s_{1})a_{0}=0$$

(26) $$\left(-\omega^{2}M-k_{3}(5a_{0}^{4}+\frac{15}{2}a_{0}^{2}\eta^{2}+\frac{5}{8}\eta^{4}\right)-(k_{2}-s_{2})(3a_{0}^{2}+\frac{3}{4}\eta^{2})-(k_{1}-s_{1})+\ldots \;(\frac{\theta^{2}\omega^{2}R^{2}C_{p}}{1+\omega^{2}R^{2}C_{p}^{2}}{)}^{2}\eta^{2}+\left(\omega C+\frac{\theta^{2}\omega^{2}R}{1+\omega^{2}R^{2}C_{p}^{2}}\right)\eta^{2}={\left(H_{s}A\right)}^{2}$$

where the amplitude of system response displacement $\eta =\sqrt{\left(a^{2}+b^{2}\right)}$, and the amplitude of system response voltage $V=\sqrt{\left(p^{2}+q^{2}\right)}$.

By solving Equations (25) and (26), the steady-state response spectrum of the system can be obtained. Generally, the harmonic solution contains stable solution and unstable, which can be judged by the Jacobian matrix [38]. The maximum (high branch) and minimum (low branch) solutions of nonlinear systems are stable, and can be verified by experiments, whereas the middle solution is unstable.

## 4. Analysis

**Figure 6:**
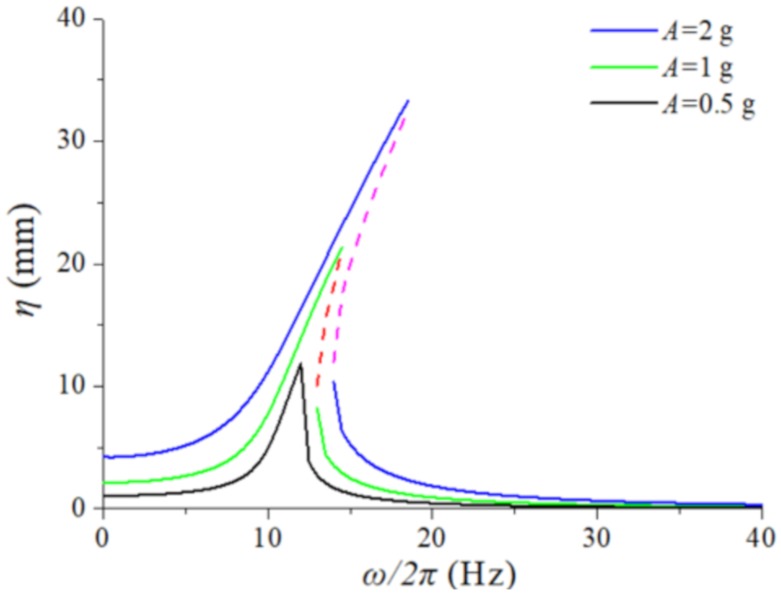
The amplitude-frequency response of the TPEH-C.

Depending on the analysis in Section 2, the restoring force of the linear-arch composite beam is nonlinear. Therefore, the dynamic response is discussed when the system does not contain magnetic force only considering the nonlinear restoring force. The spectrum response of the system is obtained by making $k_{3}=k_{2}=k_{1}=0$ in Equations (25) and (26). Select the excitation acceleration *A* = [0.5 g, 0.8 g, 1 g] to get the response spectrum under different excitation acceleration seen in Figure 6. It can be seen that the spectrum characteristic of the linear-arch composite beam at *A* = 0.5 g is similar to that of the linear system. However, as the excitation level increases, the curve gradually bends in the direction of increasing frequency, showing a harden characteristic. There are multiple solutions in a certain frequency range, where the dashed line part is unstable solution and the solid line part is stable solution. It indicates that the linear-arch composite beam is nonlinear, and the nonlinear spectrum characteristic becomes more obvious with the increase of the external excitation amplitude. Compared with the response spectrum characteristics of the linear system, the nonlinear system has a large amplitude in a wide frequency range.

**Figure 7:**
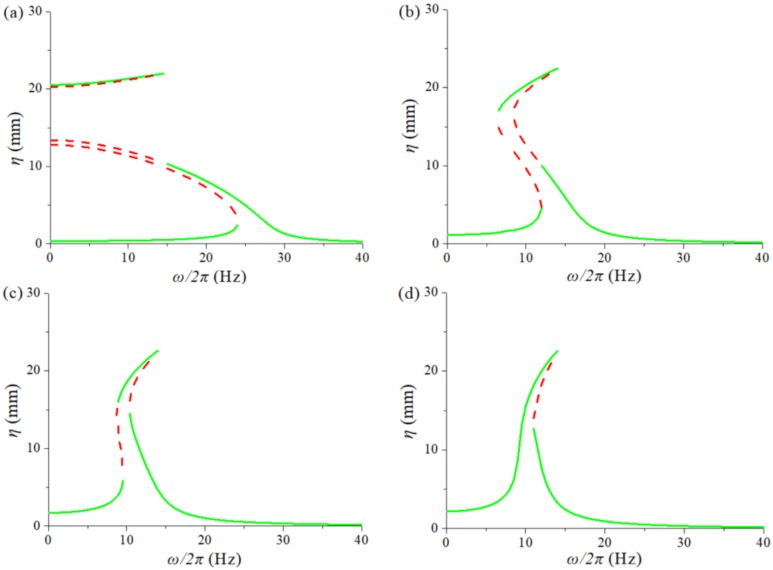
The response frequency spectrum of the TPEH-C with different magnetic distance: (**a**) *d* = 4 mm; (**b**) *d* = 8 mm; (**c**) *d* = 10 mm; (**d**) *d* = 12 mm.

Figure 7 shows the response spectrum of the TPEH-C with different magnetic distance. Select excitation *A* = 1 g, magnet distance *d* = [4 mm, 8 mm, 10 mm, 12 mm]. Figure 7a is the response spectrum of the TPEH-C at *d* = 4 mm, at which point the figure contains the high-energy interwell oscillations and the low-energy intrawell motion. When η>20 mm, the system does the interwell motion. In practice, if the barrier between wells is deep enough, the motion needs to be excited by external force or large enough external excitation. Figure 7b,c is the system response spectrum when *d* = 8 mm and *d* = 10 mm, respectively. It can be seen that the curve first bends in the low frequency direction and then in the high frequency direction, forming the “S” shape. Furthermore, a broadband with a larger response is formed when bending to the high frequency side. The bandwidth at *d* = 8 mm is about 7.5 Hz and *d* = 10 mm is about 5.5 Hz. This indicates that the nonlinear magnetic force will affect the response bandwidth of the system in a certain range of magnet spacing, and the width decreases as the magnetic distance increases. Figure 7d is the response spectrum at *d* = 12 mm. The spectrum of the system is already close to the response spectrum under the magnet-free condition illustrated in Figure 6. It indicates that the magnetic distance has reached the critical point of failure range of the magnetic force. Furthermore, if the distance exceeds the distance of 12 mm, the system is equivalent to no magnetic condition.

**Figure 8:**
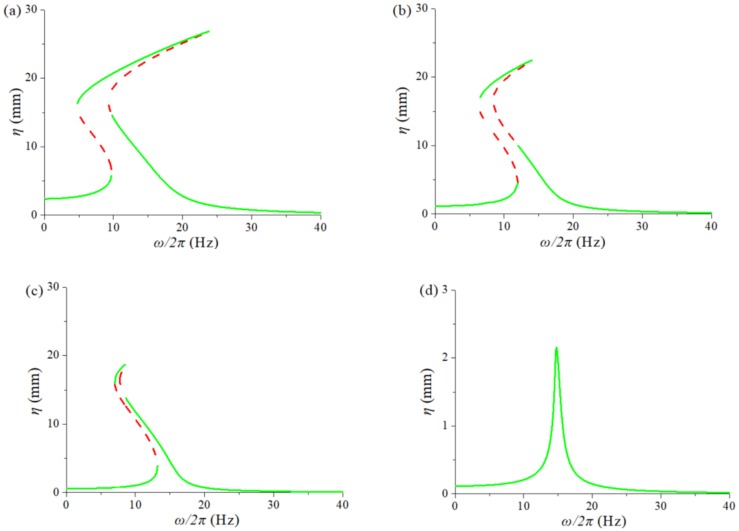
The response frequency spectrum of TPEH-C under excitation acceleration: (**a**) *A* = 2 g; (**b**) *A* = 1 g; (**c**) *A* = 0.5 g; (**d**) *A* = 0.1 g.

Figure 8 indicates the displacement response spectrum of the TPEH-C at different excitation acceleration. Select the distance of the magnet *d* = 8 mm, excitation *A* = [0.1 g, 0.5 g, 1 g, 2 g]. Figure 8a shows the system displacement response spectrum for *A* = 2 g. It can be seen that the system has a large interwell response in the range of 15~25 Hz. Whereas, with the weakening of the excitation (the excitation in Figure 8b,c are 1 g and 0.5 g, respectively), and the response band becomes narrower and the amplitude decreases. When the excitation *A* = 0.1 g, as shown in Figure 8d, the response spectrum of the system presents as the linear system, and the response amplitude is reduced by 10 times. The excitation is not enough to make the system cross the barrier, and the system does the small intrawell motion. Therefore, the magnitude of the external excitation not only affects the response amplitude of the TPEH-C, but also affects the effective frequency bandwidth.

## 5. Experimental Verification

**Figure 9:**
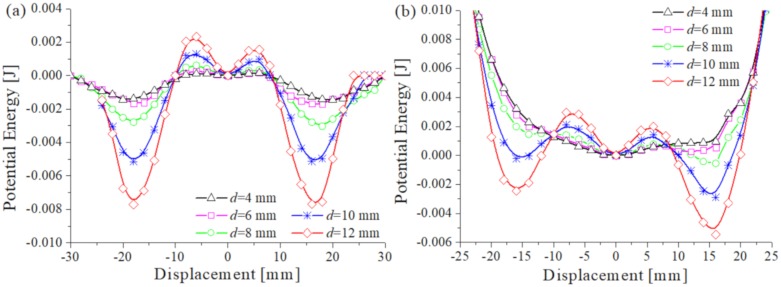
(**a**) The magnetic potential curves at different magnet distances; (**b**) the nonlinear restoring force potential curves of the TPEH-C.

In this section, the potential energy values of the TPEH-C in each position are obtained by using the experimental results of the magnetic force and the restoring force, and the potential energy-displacement curve is plotted in Figure 9. Figure 9a shows the magnetic potential energy at different magnet distances, and Figure 9b is the nonlinear restoring force potential energy of the TPEH-C. Compared with the potential energy-displacement curve obtained from the theoretical analysis, the basic law of experiment results is consistent. That is, as the magnet distance *d* increases, the potential well depth gradually decreases. The restoring force of the composite beam shallows the depth of the potential well on both sides of the TPEH-C and deepens the depth of the potential well in the middle. In addition, another new effect has been found. The potential wells in Figure 9b show asymmetry due to the unsymmetrical of the experimental results of the composite beam restoring forces, which is mentioned in Section 2.2.

**Figure 10:**
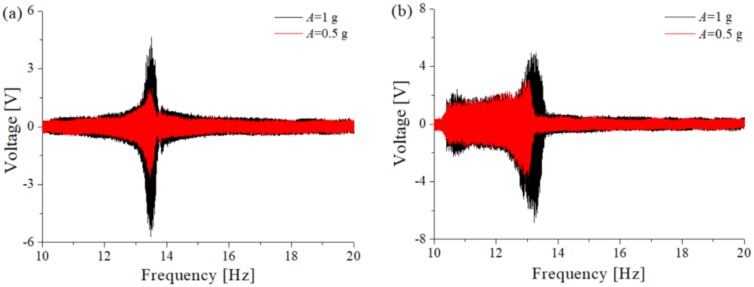
The sweep voltage diagram under two excitations: (**a**) no magnet; (**b**) TPEH-C.

In checking to see the correctness of the dynamic analysis, the experiment was conducted, which is consist of digital oscilloscope, multi-channel vibration controller, power amplifier, computer, vibration exciter and the TPEH-C. The response of linear-arch composite beam with no magnet is tested. Select excitation acceleration *A* = [0.5 g, 1 g], and the sweep frequency obtains the frequency-voltage diagram shown in Figure 10a. It can be observed that the variation of output voltage with frequency is consistent with the simulation results. The system presents nonlinear hard characteristic and the bandwidth becomes wider with the increase of excitation amplitude. The output positive and negative voltage of the system is asymmetrical, which is caused by the deformation of the piezoelectric layer is asymmetrical due to the horizontal and longitudinal deformation of the arch structure during the deformation process. Figure 10b selects the magnetic distance *d* = 10 mm to contrast the response of the system under two external excitation conditions. It shows that the magnitude of the external excitation will affect the amplitude and bandwidth of the system response. As the excitation increases, the amplitude of the system response increases and the frequency band becomes wider.

**Figure 11:**
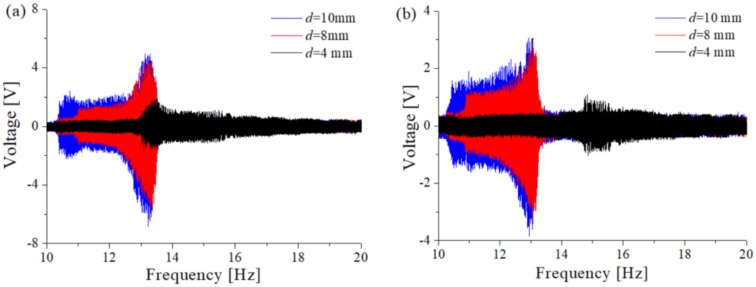
The sweep voltage diagram with different magnetic distances: (**a**) *A* = 1 g; (**b**) *A* = 0.5 g.

Select the magnetic distance *d* = [4 mm, 8 mm, 10 mm] to test the output voltage of TPEH-C when the excitation a is 1 g and 0.5 g, respectively. Figure 11a is the output voltage sweep of external excitation *A* = 1 g and Figure 11b shows the result at *A* = 0.5 g. It can be seen that it is consistent with the simulation results. When the magnetic distance is smaller, the system presents a small intrawell motion, and the response output voltage is smaller. The peak voltage in Figure 10a is about 1.8 V and in Figure 11b is about 1 V. As the distance of the magnet increases, the tri-stable barrier becomes shallower and the system moves across different potential energy wells. The system output voltage reaches a maximum of 5 V in Figure 11a and 3 V in Figure 11b.

**Figure 12:**
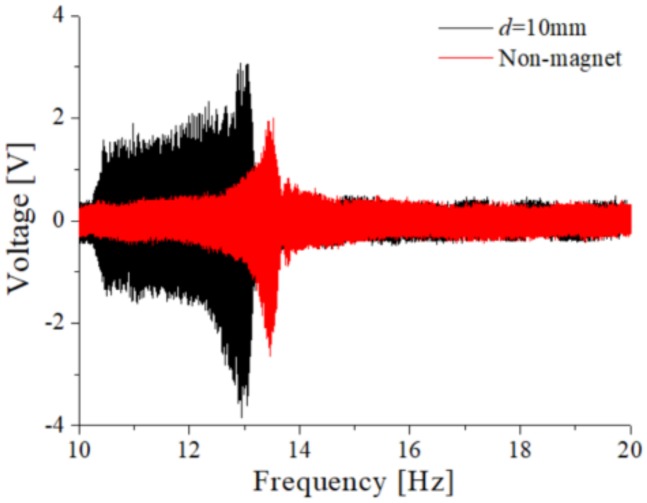
Comparison of output voltage between TPEH-C and non-magnetic system.

Figure 12 compares the output voltages of the TPEH-C and the non-magnetic harvester (monostable one) under *A* = 0.5 g. The base acceleration for Figure 12 is same, which is realized by using the controlling system to make the shaker output some excitation. The frequency band of a magnet-free system is narrow about 1 Hz, while that of a TPEH-C is about 3 Hz. This means the bandwidth of the TPEH-C is wider than the non-magnetic harvester. Therefore, the TPEH-C achieves broadband characteristics frequency.

## 6. Conclusions

In this paper, a distributed parameter model of the tri-stable piezoelectric energy harvester for linear-arch composite beam is established. By solving the harmonic balance solution of the system dynamics equation, the nonlinear response characteristics are discussed. The influence of different magnetic distances and different external excitations on the response of the TPEH-C is revealed. Finally, the numerical results are verified by experiments, and the following conclusions can be made.

(a) The arched part of the composite beam makes the cantilever be a nonlinear structure. The existence of the nonlinear restoring force makes the response spectrum curve of the composite beam bend in the direction of increasing frequency, showing the hard characteristic. Furthermore, the restoring force of the composite beam also influences the potential well depth of the tri-stable system, which could be a new way to ameliorate the potential well depth in cases where size is limited.

(b) Changing the magnet distance enables the system to switch between tri-stable and monostable states, and the appropriate magnet distance d enables the system to achieve a “wideband”. Different *d* needs to be considered in the design of the TPEH-C, which will make the response expand to the low frequency. Therefore, the design of the composite beam can extend to the high frequency according to the actual vibration source characteristic.

(c) Response bandwidth and amplitude of the TPEH-C are affected by external excitations. The frequency bandwidth of different magnet distances under the same excitation is changed, therefore, the optimal magnet distance corresponding to different excitation range should be considered in design.
